# Distribution of soil macrofauna across different habitats in the Eastern European Alps

**DOI:** 10.1038/s41597-022-01717-4

**Published:** 2022-10-18

**Authors:** Julia Seeber, Michael Steinwandter, Erich Tasser, Elia Guariento, Thomas Peham, Johannes Rüdisser, Birgit C. Schlick-Steiner, Florian M. Steiner, Ulrike Tappeiner, Erwin Meyer

**Affiliations:** 1 Institute for Alpine Environment, Eurac Research, Viale Druso 1, 39100 Bozen/Bolzano, Italy; 2 Department of Ecology, University of Innsbruck, Technikerstrasse 25/Sternwartestrasse 15, 6020 Innsbruck, Austria

**Keywords:** Biodiversity, Ecology

## Abstract

Macro-invertebrates are important components of soil ecosystems as they provide a wide range of crucial functions and ecosystem services. Knowledge on their distribution in mountain soils is scarce despite the importance of such soils for people living in mountain regions as well as downstream. The present dataset contains records on soil macro-invertebrates belonging to nineteen taxa listed at class or order level and earthworms listed at species level from 22 different habitat types characteristic for the Eastern European Alps. Data were collected over a period of more than 30 years (1987–2020) following a standard protocol. We compiled 1572 single records from 241 unique sampling sites, providing default site parameters (GPS coordinates, habitat type, type of management, elevation, exposition, inclination, bedrock, soil type following WRB classification). Such data are important to analyse global trends and macroecological patterns and to set a basis for tracking long-term changes in macrofauna composition. In addition, this dataset will add to the still sparse knowledge on the occurrence and abundance of alpine soil fauna taxa.

## Abstract (summary)

**Table:** 

Measurement(s)	abundances of soil animals
Technology Type(s)	soil samples
Factor Type(s)	NONE
Sample Characteristic - Organism	Lumbricidae • Arthropoda • Mollusca
Sample Characteristic - Environment	Alpine habitats
Sample Characteristic - Location	European Alps

## Background & Summary

Soil macro-invertebrates are animals which inhabit different soil layers, including litter and soil surface, and which are visible to the naked eye, with a body length of >1 cm and a body width >2 mm^1^. Taxonomically, they are heterogeneous and predominantly belong to the phyla Mollusca, Annelida, and Arthropoda. Their effect on the soil ecosystem is indispensable, they physically alter the soil matrix by creating burrows which increases aeration and drainage, they promote litter decomposition, and by interacting with microorganisms, they contribute to nutrient cycling^2^. Macro-invertebrates living in the soil are mainly detritivores and predators, they can be classified into various functional guilds based on their feeding habits^3^, and are as such parts of extremely complex soil food webs^4^.

Soil invertebrates closely interact with their environment, mostly due to the physical closeness. They thus strongly depend on suitable soil conditions^5^ and many soil invertebrates react sensitively to pH value, soil organic matter content, and soil texture, while others, more euryoecious species, tolerate a wide range of soil conditions. Due to the direct link between plant communities and soil properties, habitat types may exhibit very different conditions for soil animals and may thus harbour disparate soil macrofauna communities^6–8^. Mountain soils as found in the Eastern European Alps are different from soils in the lowlands and in the valley bottoms^9^. They are shallow, often acidic (on silicious bedrock), and contain a high amount of coarse grain (coarse sand and stones) as well as soil organic matter due to limitations of biological activity caused by low temperatures and a short growing season^10^. These characteristics become more prominent with increasing elevation^11^. The specific features of mountain soils and the increasing shortening of the growing season with increasing elevation call for adaptations in the physiology, phenology, and behaviour of soil invertebrates^12^. Densities of soil macro-invertebrates thus decrease with increasing elevation. Many species reach their upper limit of distribution close to the Alpine treeline or soon after^13^. Data on macrofauna composition and distribution in mountain soils is, however, scarce. The availability of more data would be important to enable reliable investigation of global trends and macroecological patterns and to set a basis for tracing alterations in macrofauna composition on the long run^14^. Here, we present a dataset containing abundance data of various soil macro-invertebrate groups from 22 different natural to artificial habitat types characteristic for the Eastern European Alps.

## Methods

### Study areas

**Fig. 1:**
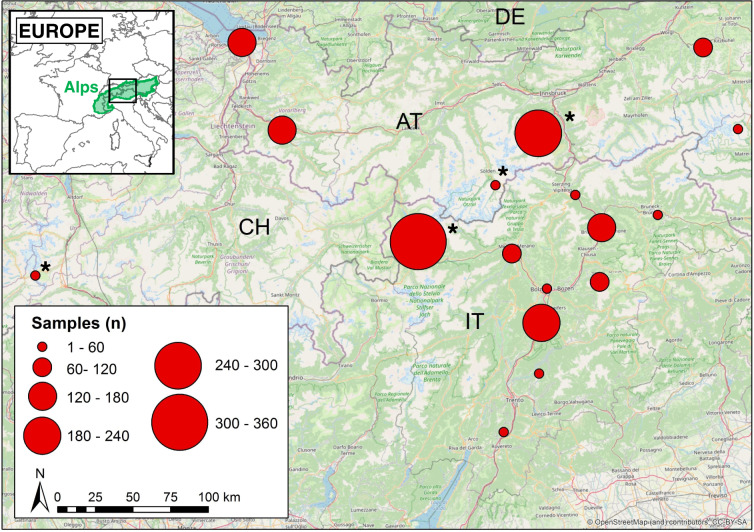
Location of study areas and number of soil fauna samples per area. Study areas may comprise more than one study site and habitat type. LTER sites are marked with an asterisk (*).

All study areas are located in the Eastern European Alps (Fig. 1), with the exception of one study site in the Western Alps (Furka, Canton of Uri, Switzerland) and one study site in the southern outskirts of the Alps (Pomarolo, Trentino, Italy). Elevation ranges from 200 to 2800 m above sea level (a.s.l.). The study sites comprise a huge variety of habitat types widely distributed in the Alps, ranging from differently managed grasslands (pastures, extensively used hay meadows, intensively used hay meadows), arable land, permanent cultures (vineyards, apple orchards), and wetlands (riparian forests, moors) to different kinds of forests (deciduous forests, coniferous forests, and shrubland).

The study areas include four Long-Term Ecological Research (LTER) sites: LTER_EU_CH_023 - Alpine Research Station Furka, ALPFOR, Switzerland; LTER_EU_IT_097 - Val Mazia/Matschertal, Italy; LTER_EU_AT_015 - Tyrolean Alps - Stubai, Austria; and LTER_EU_AT_018 - Tyrolean Alps - Obergurgl, Austria.

### Fieldwork

Even though the sampling period spans more than 30 years (1987–2020), soil macrofauna samples were taken using more or less the same method throughout with slight adaptations only. A soil fauna sampler was driven into the soil (10 to 15 cm deep, depending on soil depth and stones, especially at high alpine sites), the obtained soil sample was put into a linen bag, labelled, and transported to the soil laboratory as soon as possible, but within two days maximum. Depending on the habitat type, vegetation was cut to 1 cm from the surface, and in forests, the litter was removed. The size of the soil fauna sample depended on the sampling site; the commonly used size was 706.9 cm^2^ (sampler of ⌀ 30 cm) but was sometimes reduced to 176.7 cm^2^ (sampler of ⌀ 15 cm), mainly for (high) alpine sites due to the destructiveness of soil sampling. From 2015 onwards, sampling predominantly was done using a soil fauna sampler of 400 cm^2^ (20 × 20 cm). At the soil laboratory, invertebrates were extracted by heat in a modified Kempson apparatus^15^ for 10–14 days (depending on soil water content and clay content) until complete desiccation. As collection fluid, picric acid was used in earlier years, but from around 2001 onwards non-toxic ethylene glycol or propylene glycol was mostly used. After extraction, soil animals were rinsed with tap water in a sieve (100 µm mesh size) and transferred to 75% ethanol until further processing. Whenever possible, the dry soil was returned to its place of origin to minimise the impacts of the destructive sampling (especially at higher elevations). Voucher specimens are deposited at the University of Innsbruck or at Eurac Research.

At each sampling site, default site parameters were recorded (GPS coordinates, habitat type^16,17^, type of management, elevation, exposition, inclination, bedrock, soil type following WRB classification^18^). In earlier field studies (before 2000), the GPS coordinates were taken just once in the centre of the bigger sampling area. The exact position of these sites was reconstructed as accurately as possible, but unfortunately, not all were retrievable due to the sudden passing of the main investigator Erwin Meyer.

### Identification of soil macro-invertebrates

The animals were determined to class or order level and in case of earthworms (Lumbricidae) to species level under a dissecting microscope. The identification of Lumbricidae followed Christian and Zicsi (1999)^19^ and Czusdi and Zicsi (2003)^20^, that of all other taxa Schaefer (2009)^21^ and Klausnitzer (2011)^22^. Individuals were counted and densities (ind. m^−2^) were calculated.

Taxonomic sufficiency^23^, the concept of identifying organisms at a satisfactory level for the study in question, has been widely discussed. Identifying soil macro-invertebrates to a higher taxonomic resolution is time-consuming and needs taxonomic expertise for many different groups^24^. Therefore, a low taxonomic resolution has been the norm in soil ecological and zoological research for a long time and it has been shown to be sufficient to identify general patterns for entire communites^24–27^ or individual taxa^28^. Commonly used biodiversity indices such as the QBS index^29^ or the IBQS index^30^ work with low taxonomic resolution. However, for specific research questions, for example for modelling food webs^4^, a higher taxonomic resolution is necessary. As in the 80s and 90s such high taxonomic resolution was still the exception, we had to stick to the low taxonomic resolution to create a uniform dataset, but for samples taken after 2000, data in high taxonomic resolution (e.g. diplopods on family or even species level, Coleoptera and Diptera larvae on family level) is available upon request or already accessible on the digital data library PANGAEA^®^ (see Table 1).

**Table 1:** Dataset overview listing the single soil macro-invertebrate samplings with their associated publications and published datasets on the data repository PANGAEA^®^.

Rows	Region	Publication	Dataset
1–71	Nature Reserve Rheindelta, Vorarlberg, AT	Meyer, A. & Steinberger, K.H. Die Spinnenfauna des Naturschutzgebietes Rheindelta (Vorarlberg, Österreich) (Arachnida: Araneae). *Berichte des naturwissenschaftlichen-medizinischen Vereins Innsbruck* 82, 195–215 (1995)^34^.	
72–108	South Tyrol & Trentino, IT	Kopeszki, H. & Meyer, E. Artenzusammensetzung und Abundanz von Collembolen in Waldböden der Provinzen Bozen und Trient (Italien). *Berichte des naturwissenschaftlichen-medizinischen Vereins Innsbruck* 83, 221–237 (1996)^35^.	
109–183	Brixen im Thale, Tyrol, AT	Geitner, C., Mätzler, A., Bou-Vinalis, A., Meyer, E. & Tusch, M. Soil characteristics and colonization by earthworms (Lumbricidae) on pastures and hay meadows in the Brixenbach valley (Kitzbühel Alps, Tyrol). *Die Bodenkultur* 65(1), 39–51 (2014)^36^.	
184–249	Stubai Valley, Tyrol, AT	Kössler, W. Die Makrofauna in Almböden unter Berücksichtigung der Landnutzung und des Gesteinsuntergrundes im Bereich der Kaserstattalm oberhalb von Neustift im Stubaital (1.860–2.170 m NN). *Diploma Thesis*, pp. 71, University of Innsbruck, Austria (2001).	
250–308	Stubai Valley, Tyrol, AT	Neyer, A. Abundanz und Biomasse der Makrofauna in alpinen Böden im Bereich der Kaserstattalm oberhalb von Neustift im Stubaital (2.170–2.600 m ü. M.). *Diploma Thesis*, pp. 133, University of Innsbruck, Austria (2001).	
309–542	Vorarlberg, AT	Meyer, E. & Steinberger, K.H. Über die Bodenfauna in Wäldern Vorarlbergs (Österreich) – Bestand und Auswirkungen von Gesteinsmehlapplikationen. *Verhandlungen der Gesellschaft für Ökologie* 23, 149–164 (1994)^37^.	
543–682	South Tyrol, IT	Peham, T. & Meyer, E. Kommentierte Artenlisten ausgewählter Bodentiergruppen aus der Erhebung des SoilDiv-Projektes in Südtirol. *Gredleriana* 14, 227–262 (2014)^38^.	
683–714	South Tyrol, IT	Rottensteiner, M. Bodenmakrofauna auf Ackerflächen in Südtirol. *Master Thesis*, pp. 103, University of Innsbruck, Austria (2020).	
715–786	Stubai Valley, Tyrol, AT	Steinwandter, M., Rief, A., Scheu, S., Traugott, M. & Seeber, J. Structural and functional characteristics of high alpine soil macro-invertebrate communities. *Eur. J. Soil Biol*. 86, 72–80 10.1016/j.ejsobi.2018.03.006 (2018)^39^.	
787–842	Stubai Valley, Tyrol, AT	Steinwandter, M., Schlick-Steiner, B. C., Seeber, G. U. H., Steiner, F. M. & Seeber, J. Effects of Alpine land-use changes: Soil macrofauna community revisited. *Ecol. Evol*. 7, 5389–5399 10.1002/ece3.3043 (2017)^25^.	
843–862	Martell Valley, South Tyrol, IT	Schwembacher, A. Arthropoden im Hochgebirge - eine Untersuchung der epigäischen Fauna im hinteren Martelltal. *Master Thesis*, pp. 137, University of Innsbruck, Austria (2015).	
863–880	Stubai Valley & Obergurgl, both Tyrol, AT	Seeber, J. & Steinwandter, M. – unpublished	
881–912	Kaltern/Caldaro, South Tyrol, IT	Steinwandter, M. *et al*. Does green manuring positively affect the soil macro-invertebrates in vineyards? A case study from Kaltern/Caldaro (South Tyrol, Italy). *Gredleriana* 18, 17–26 (2018)^40^.	Steinwandter *et al*.^41^
913–1056	LTSER Matsch Valley, South Tyrol, IT	Damisch, K., Steinwandter, M., Tappeiner, U. & Seeber, J. Soil macroinvertebrate distribution along a subalpine land use transect. *Mt Res Dev* 40, 10.1659/MRD-JOURNAL-D-19-00057.1 (2020)^42^.	Damisch *et al*.^43^
1057–1164	LTSER Matsch Valley, South Tyrol, IT	Seeber, J. & Steinwandter, M. – unpublished	
1165–1191	LTSER Matsch Valley, South Tyrol, IT	Schneider, E., Steinwandter, M. & Seeber, J. A comparison of Alpine soil macroinvertebrate communities from European larch and Swiss pine forests in the LTSER area “Val Mazia/Matschertal”, South Tyrol. *Gredleriana* 19, 217–228 10.5281/zenodo.3565374 (2019)^44^.	Schneider *et al*.^45^
1192–1245	Central European Alps	Seeber, J. *et al*. Soil invertebrate abundance, diversity, and community composition across steep high elevation snowmelt gradients in the European Alps. *Arct. Antarct. Alp. Res.* 53, 288–299 10.1080/15230430.2021.1982665 (2021)^46^.	Seeber *et al*.^47^
1246–1527	South Tyrol, IT	Guariento, E. *et al*. Management Intensification of Hay Meadows and Fruit Orchards Alters Soil Macro- Invertebrate Communities Differently. *Agronomy* 10, 767 10.3390/agronomy10060767 (2020)^48^.	
1528–1573	Obergurgl, Tyrol, AT	Enderle, E. *et al*. Suitability of different taxonomic resolutions in environmental monitoring: case study soil macrofauna along an alpine transect. (in preparation).	

## Data Records

We uploaded the dataset “A 30-years collection of soil macro-invertebrate abundance data from the European Alps^31^.” to PANGAEA^®^ Data Publisher, from where it can be downloaded as tab-delimited text (.csv) or as original Microsoft^®^ Excel file (.xlsx). The dataset comprises abundance data of four taxa on class level (Chilopoda, Symphyla, Pauropoda, Diplopoda), fifteen taxa on order or suborder level (Gastropoda, Araneae, Pseudoscorpiones, Opiliones, Isopoda, Protura, Diplura, Thysanoptera, Dermaptera, Homoptera, Heteroptera, Coleoptera, Lepidoptera larvae, Diptera larvae), and seventeen earthworm species, with 1572 single records from 241 unique sampling sites. Also included are data on site characteristics (GPS coordinates, description of the location, habitat type, type of management, elevation, exposition, inclination, bedrock, soil type).

## Technical Validation

Quality assurance during the field and laboratory work was provided by Erwin Meyer, Michael Steinwandter and Julia Seeber by overseeing field sampling and reviewing the final data tables. All data entries were meticulously checked for any incongruencies and spelling mistakes by the authors JS, MS, and ET before upload to PANGAEA^®^ Data Publisher, where it was then reviewed by a data curator before assigning a DOI. Taxonomic nomenclature follows Fauna Europaea^32^.

### Data content

For a first overview of the data, two biodiversity indicators were calculated: a) group richness (number of taxa on class, order or suborder level per single record), and b) Shannon diversity index (SHDI)^33^. The statistical analyses were carried out with IBM SPSS Statistics 27.

**Fig. 2:**
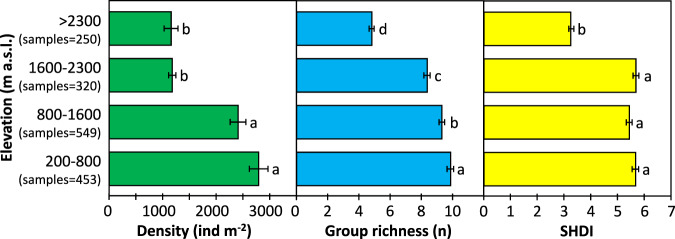
Diversity changes in soil macro-invertebrates with elevation. Post-hoc pair-wise comparisons were performed using Bonferroni tests. Values with different letters indicate significant difference between the means at significance level *p* < 0.05. SHDI… Shannon Diversity Index.

The samples contained in the dataset reveal clear trends. A decrease in diversity of soil macro-invertebrates with an increase in elevation is evident (Fig. 2). On average, individual densities decrease by more than 60% in the high montane/subalpine belt (sites above 1600 m) compared to lower sites. Group richness decreases steadily with elevation, while the Shannon Diversity Index (SHDI) decreases significantly only after the transition from the subalpine to the alpine belt (sites above 2300 m).

The distribution and diversity of soil macro-invertebrates is also strongly influenced by habitat type and land use. Our data show that on one hand natural alpine grasslands are in general less diverse than habitats in lower elevation (Table 2), with the limitation that except for earthworms, no data on species level are included. Forests, on the other hand, are the most diverse habitats in terms of individual density, group richness, and SHDI. Within forests, the high-elevation types (knee timber) and wet forests show below-average group richness and SHDI, while thermophilic deciduous forests of the colline belt show particular above-average group richness. Agricultural use in all forms leads to a decrease in individual density, group richness and SHDI, whereby agroforestry systems are more diverse regarding group richness and SHDI than all other agricultural systems. Agricultural habitat types on special sites (xeric grassland, moors/wet meadow) and at higher elevations (alpine pastures), and especially arable farmland, are less diverse. The very high density of individuals and richness of groups in green spaces in settlements is also remarkable.

**Table 2:** Descriptive density and diversity of soil macro-invertebrates in different habitat types with information about their mean elevation distribution.

Habitat type	Elevation [m a.s.l.]	Samples [n]	Density [ind. m^−2^]		Groups [n]		SHDI	
			$\bar{x}$	s.d.	$\bar{x}$	s.d.	$\bar{x}$	s.d.
1 Alpine grasslands	2254	179	1193.1	337.4	8.2	0.8	5.6	0.4
1.1 Dwarf shrub communities, limestone	2492	28	1061.0	470.2	9.4	1.4	7.2	1.2
1.2 Natural alpine grassland, limestone	2440	67	1024.8	937.9	7.8	2.9	5.1	2.2
1.3 Dwarf shrub communities, silicate	2134	37	1648.3	1417.8	9.0	3.5	5.8	2.2
1.4 Natural alpine grassland, silicate	1950	47	1038.2	1033.5	6.7	3.0	4.3	1.8
2 Forests	1083	368	4618.5	1139.0	12.0	0.9	6.0	0.5
2.1 Knee timber	1934	10	2291.8	1285.4	9.8	3.0	4.6	1.3
2.2 Sub-alpine coniferous forest	1940	36	4214.9	3839.5	11.1	4.2	5.7	1.5
2.3 Montane spruce-fire forest	1148	231	3240.1	4463.8	11.1	3.0	6.0	2.1
2.4 Pine forest	751	14	6124.7	4140.7	13.4	2.6	6.4	2.5
2.5 Mixed forest	862	41	3112.5	2042.0	14.5	1.6	9.0	1.3
2.6 Thermophile oak forest	550	12	10250.1	4344.0	15.1	1.4	5.2	1.1
2.7 Wet forest	400	24	3095.9	3221.3	9.4	2.3	4.9	2.2
3 Agroforestry systems	1250	125	1862.7	152.6	9.6	0.6	6.4	0.2
3.1 Larch meadow	1719	53	1972.6	1681.7	9.7	3.4	6.6	2.2
3.2 Orchard meadow	781	72	1752.8	1376.4	9.5	2.3	6.2	1.9
4 Extensively used agricultural areas	1221	524	1410.5	388.8	7.4	0.4	4.6	0.2
4.1 Rough meadow, alpine pastures	2128	311	1152.2	1717.7	5.9	2.9	3.9	2.0
4.2 Xeric grassland	1321	72	1060.8	941.9	6.7	2.4	4.2	1.7
4.3 Semi-rough meadow	1034	105	1697.9	1897.9	9.1	2.7	6.2	1.9
4.4 Moors/wet meadow	400	36	1731.1	1862.6	7.8	1.9	4.0	1.6
5 Intensively used agricultural areas	738	366	1878.4	1231.2	7.6	0.9	4.6	0.3
5.1 Fodder meadow	974	156	2293.7	2228.1	8.6	2.8	5.1	1.7
5.2 Arable farmland	994	42	697.1	779.9	5.4	1.5	3.3	1.4
5.3 Fruit plantation	608	106	2078.9	3013.1	8.7	3.1	5.3	1.8
5.4 Vineyard	376	62	2444.1	4166.3	7.8	4.1	4.6	2.2
6 Green space in settlements	348	10	5797.5	4921.4	10.7	2.8	4.4	1.8
Mean (all)	1253	1572	2076.9	2904.7	8.6	3.6	5.2	2.2

SHDI… Shannon Diversity Index.

The soil macrofauna data and associated environmental data also allow analyses of the effects of topographic factors, management, and soil indicators on soil macro-invertebrates. In addition, the data collection over a period of 34 years (1987–2020) enables an analysis of diversity changes over time, even though this might be limited due to heterogenous distribution of samples within habitat types over time (Table 3). For forests, intensively and extensively used agricultural areas, sampling data are available over the entire period. For alpine habitats, data are available since 1995 (over 3 time periods). For settlements and agroforestry systems, however, no statements can be made due to missing time series.

**Table 3:** Distribution of soil fauna samples over time (1987–2020).

	1985–1995	>1995–2005	>2005–2015	>2015–2020	Sum
Alpine grasslands	0	94	55	30	179
Forests	262	8	44	54	368
Agroforestry systems	0	8	0	117	125
Extensively used agricultural areas	36	23	183	282	524
Intensively used agricultural areas	11	0	120	235	366
Green space in settlement	0	0	10	0	10
Sum	309	133	412	718	1572

## Data Availability

No custom code has been used to generate or process this dataset.
